# Diagnostic Diplopia: Isolated Oculomotor Nerve Palsy With Normal Imaging

**DOI:** 10.7759/cureus.115972

**Published:** 2026-09-08

**Authors:** Andrew Garfield, Andrew Dai, Chelsea McNamara, Andrew Ajemian

**Affiliations:** 1 Ophthalmology, Cooper University Hospital, Camden, USA; 2 Internal Medicine, Inspira Health Network, Mullica Hill, USA

**Keywords:** computed tomography, cranial nerve, cranial nerve iii palsy, magnetic resonance imaging, ophthalmoplegia

## Abstract

A 51-year-old man presented with progressive, subacute diplopia and ophthalmoplegia. Examination by the hospitalist demonstrated right ophthalmoplegia on left lateral gaze. No neurologist or neuro-ophthalmologist was available. Imaging and laboratory work were largely unremarkable. Although a microvascular etiology was suspected, the unremarkable workup expanded the differential diagnosis, highlighting the diagnostic and management challenges of cranial nerve III palsy when imaging is unremarkable in a community hospital with limited resources.

## Introduction

Cranial nerve III palsies have been historically well described throughout the literature [1]. The classic presentation involves ischemic and compressive subtypes, the former presenting with ptosis, a “down and out eye,” or one with impaired adduction, depression, and sparing of the pupil, while the latter demonstrates similar extraocular dysfunction but with a mydriatic, poorly reactive pupil. Etiologies of ischemic cranial nerve III palsy involve manifestations of microvascular disease, often stemming from diabetes or hypertension. Compressive etiologies classically include aneurysm of the posterior communicating artery, mass effect from intracranial lesions (e.g., meningiomas), or the cavernous sinus.

Less commonly, other causes of cranial nerve III palsies may include orbital cellulitis, Tolosa-Hunt syndrome, or idiopathic cases [2-4]. In orbital cellulitis, inflammation, edema of the orbital cavity, or direct inflammation of the extraocular muscles can cause ophthalmoplegia, which may mimic cranial nerve III palsy; however, mass effect from an abscess may spread into the cavernous sinus or orbital apex, directly resulting in a cranial nerve III palsy, sometimes with pupil involvement [1,2]. Tolosa-Hunt syndrome is a rare granulomatous disease characterized by inflammation of the cavernous sinus, orbital apex, or superior orbital fissure, resulting in a unilateral periorbital headache with potential involvement of surrounding neurovascular structures, including cranial nerves III, IV, V, and VI and the ophthalmic artery [3]. Idiopathic cases are considered diagnoses of exclusion after compressive, inflammatory, vascular, infectious, and traumatic etiologies are ruled out through comprehensive histories, examinations, and imaging [5].

Investigations and management depend on the etiology, but most investigations are thorough. Magnetic resonance imaging is essential to rule out compressive and vascular causes [6]. In Tolosa-Hunt syndrome, oral and intravenous corticosteroids are often used [3]. Blood cultures, cerebrospinal fluid analysis, and potential drainage are necessary for infectious causes, depending on dissemination and abscess formation [7]. In idiopathic cases, oral steroids have been attempted in a few case reports. Some idiopathic cases are self-limited, while others persist [4]. In cases of negative magnetic resonance imaging, the differential diagnosis can be more challenging, raising the need for a higher index of suspicion for atypical cases.

## Case presentation

A 51-year-old man presented with progressive blurred vision and vertigo for one week. He was not adherent to his blood pressure and diabetes medications. He complained of left lateral diplopia and blurry vision associated with eye movements. He had been self-patching his right eye to resolve the diplopia. He denied any recent trauma, headache, nausea, vomiting, chest pain, dyspnea, palpitations, lightheadedness, syncope, slurred speech, difficulty finding words, or motor or sensory deficits.

Vital signs were significant for slight hypertension but were otherwise normal. He remained in no acute distress. On physical examination, the pupils were equal, round, and reactive to light and accommodation bilaterally. There was significant horizontal diplopia on left lateral gaze (Figure 1), with an inability of the right eye to adduct past the midline and vertical diplopia at peak upward and downward gaze. Extraocular movements were otherwise intact bilaterally. The remaining neurological examination was unremarkable. 

**Figure 1:**
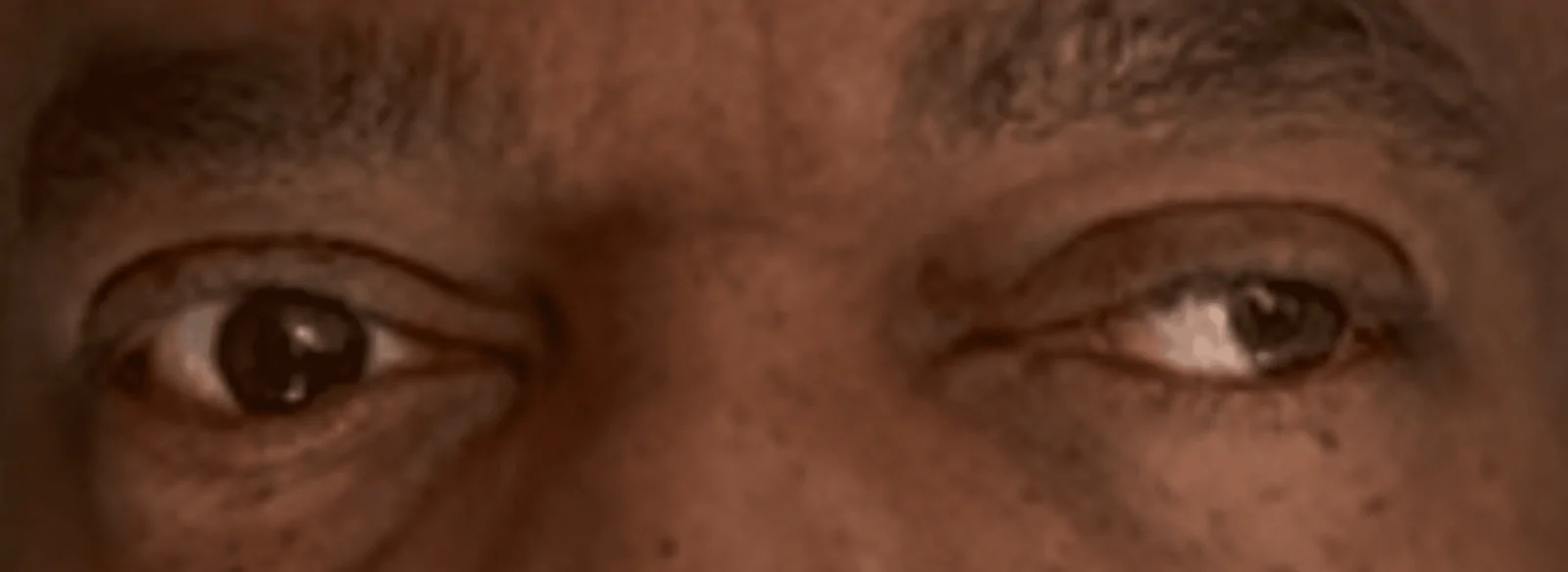
Physical examination on left lateral gaze demonstrating inability of the right eye to adduct past the midline.

Computed tomography of the head showed no acute intracranial hemorrhage, mass effect, large vessel aneurysm, occlusion, or dissections within the cerebral vasculature (Figure 2). Axial T2-weighted fluid-attenuated inversion recovery (FLAIR) magnetic resonance imaging without contrast showed no acute intracranial abnormality or mass effect (Figure 3). Findings otherwise included mild ethmoid, frontal, and maxillary sinus mucosal thickening. Laboratory studies were significant for leukopenia at 3,500 white blood cells/mm³ (reference range: 4,500-11,000/mm³), total cholesterol of 164 mg/dL (reference range: <200 mg/dL), triglycerides of 52 mg/dL (reference range: <150 mg/dL), high-density lipoprotein of 50 mg/dL (reference range: >40 mg/dL), low-density lipoprotein of 101 mg/dL (reference range: <100 mg/dL), hemoglobin A1c of 5.6% (reference range: <5.7%), vitamin B12 level of 720 pg/mL (reference range: 200-900 pg/mL), and folate level of 12.51 ng/mL (reference range: 5-20 ng/mL). A Lyme polymerase chain reaction was negative. 

**Figure 2:**
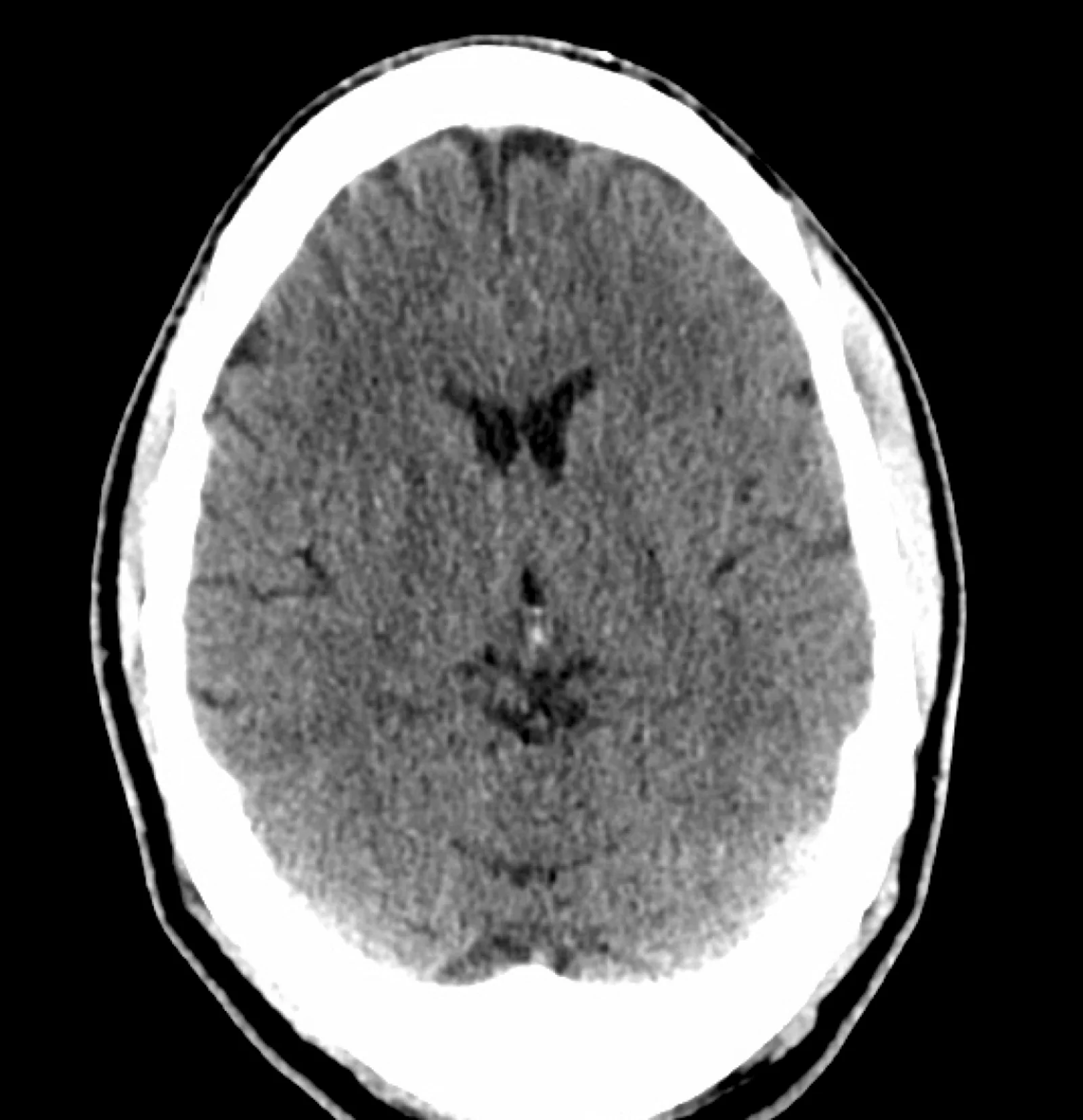
Non-contrast computed tomography showing no large vessel occlusion, hemorrhage, mass effect, or dissections.

**Figure 3:**
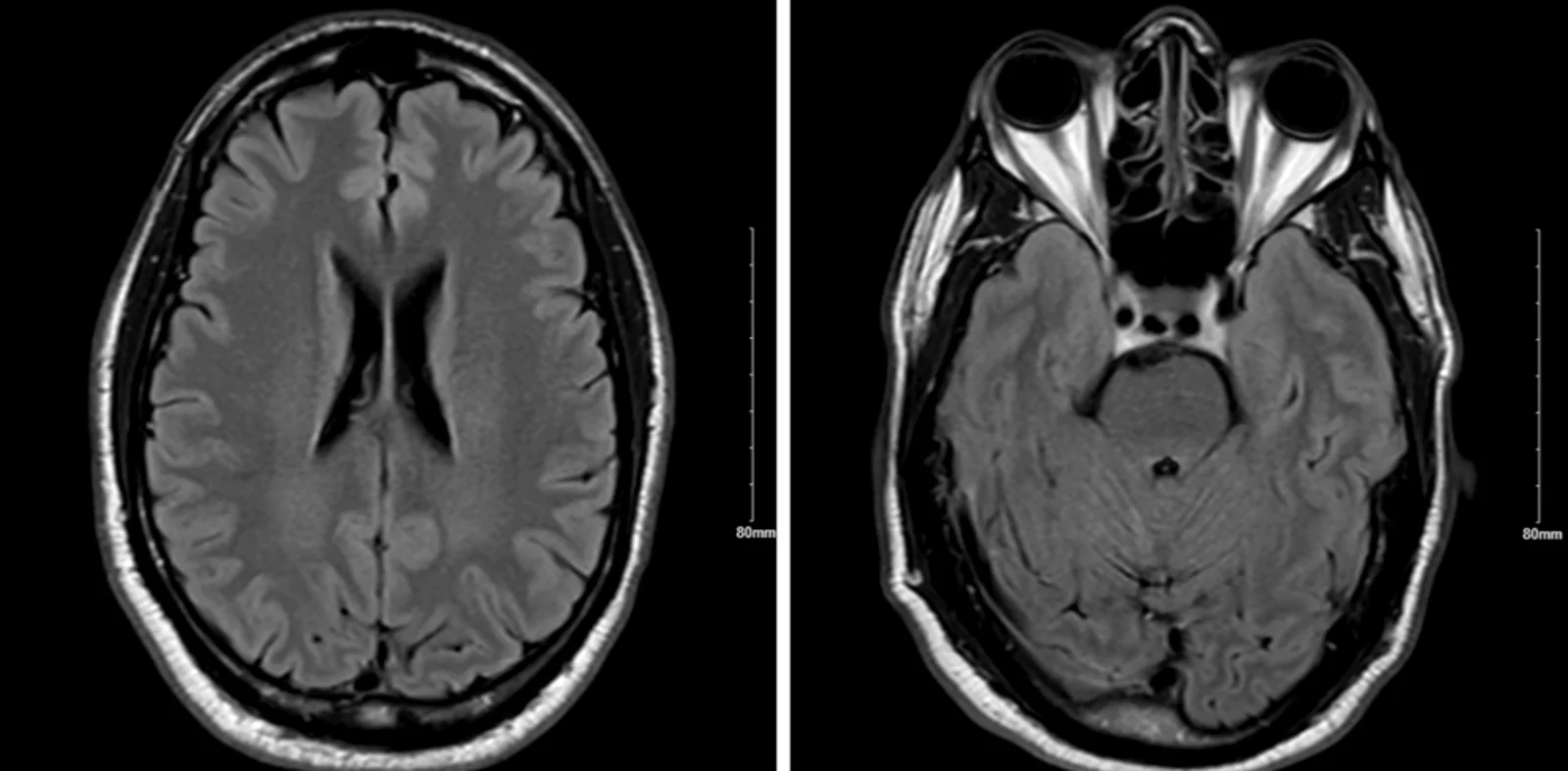
Axial T2-weighted fluid-attenuated inversion recovery (FLAIR) magnetic resonance imaging without contrast of the brain, shown in the first image, and orbits, shown in the second image, demonstrating no evidence of acute intracranial abnormality.

## Discussion

While the leading diagnosis was likely microvascular in the setting of missing hypertensive and diabetic medications, the unremarkable magnetic resonance imaging findings make this case more reassuring. The lack of vascular findings would suggest that a macrovascular ischemic etiology is less likely; however, it does not rule out a microvascular ischemic cause, such as in a patient like ours with uncontrolled hypertension.

Numerous studies suggest that the negative predictive value of magnetic resonance imaging by experienced neuroradiologists for ischemic cranial nerve III palsy is around 90%, demonstrating the possibility of a false negative, which remained in our differential for this patient given his history of medication noncompliance [6,8,9]. Even if this patient did not suffer from an ischemic cause, this case highlighted the myriad other etiologies, including idiopathic causes, that were required to be ruled out.

The neurologic impression was a suspected microvascular etiology due to horizontal diplopia present at left lateral gaze and vertical diplopia present at peak upgaze and downgaze. He was discharged with a right eye patch and plans to follow up with an outpatient ophthalmologist and was given a refill of his hypertensive and diabetic medications.

The patient was called for follow-up and noted improvement in his diplopia. He resumed his prior hypertensive and diabetic medications and is continuing to follow up with outpatient ophthalmology.

Cranial nerve palsies have long been well described in the medical literature [1]. Despite this, every case remains unique and requires independent evaluation. Typical etiologies may not always be the cause of cranial nerve palsies, such as in the patient detailed in this case report. While the etiology of isolated cranial nerve palsy in the setting of known vascular risk factors and unremarkable imaging is likely microvascular [10], in community hospitals, which often lack additional resources, such as testing or specialists, proper diagnosis and management are critical. In these settings, the hospitalist is often the only supervising physician immediately equipped to handle such cases [11]. In this case, misdiagnosis as a stroke requiring fibrinolytics or an autoimmune disease requiring alternative therapy may be devastating to the patient [12].

## Conclusions

The initial evaluation of a patient with diplopia should involve a comprehensive neurological examination. In hospitals with limited resources, ensuring up-to-date knowledge of stroke mimics is crucial to prevent misdiagnosis and improper management. Additionally, timely intervention ensures appropriate counseling, and a follow-up plan may assist in preventing similar presentations in the future.

These findings ultimately expand upon the current literature's assessment of stroke-like symptoms in community hospitals and emphasize the need for heightened clinical suspicion for proper, efficient management of isolated cranial nerve palsy.
